# Osteopathy in the Early Diagnosis and Management of Degenerative Cervical Myelopathy: National Survey

**DOI:** 10.2196/45248

**Published:** 2023-05-09

**Authors:** Jamie F M Brannigan, Oliver D Mowforth, Matthew Rogers, Helen Wood, Zahabiya Karimi, Mark R N Kotter, Benjamin M Davies

**Affiliations:** 1 Division of Neurosurgery Department of Clinical Neurosciences University of Cambridge Cambridge United Kingdom; 2 Institute of Osteopathy Luton United Kingdom; 3 Myelopathy.org Cambridge United Kingdom

**Keywords:** cervical cord, myelopathy, spondylosis, stenosis, disc herniation, ossification posterior longitudinal ligament, degeneration, disability, diagnosis, degenerative cervical myelopathy, spine, osteopathy, neurodegenerative condition, surgical decompression, neurodegeneration, survey

## Abstract

**Background:**

Degenerative cervical myelopathy (DCM) is a common and disabling neurodegenerative condition. Surgical decompression is the only evidence-based treatment to halt disease progression; however, diagnosis and access to timely treatment are often delayed, which contribute to significant disability and dependence. Supporting early diagnosis and access to timely treatment is a critical priority. Exploring these challenges, Myelopathy.org has observed that people with DCM may seek osteopathy care for their symptoms, both before and after diagnosis.

**Objective:**

This study aimed to describe the current interaction between osteopaths and people with DCM and understand how this may be targeted to enhance the DCM diagnostic pathway.

**Methods:**

Registered osteopaths in the United Kingdom completed a web-based survey hosted by the Institute of Osteopathy, as part of their institute’s 2021 census. Responses were collected from February to May 2021. Demographic information about the respondents was captured, including age, gender, and ethnicity. Professional information captured included the year of qualification; region of practice; type of practice; and number of undiagnosed, operated diagnosed, and unoperated diagnosed DCM cases encountered per year. The completion of the survey was voluntary; however, a prize draw incentive was offered to participants.

**Results:**

The demographics were heterogenous for the 547 practitioners who completed the survey. There was representation from a wide range of demographic groups, including the level of experience, gender, age, and the region of United Kingdom. At least 68.9% (377/547) of osteopaths reported encounters with DCM each year. Osteopaths most frequently encountered patients with undiagnosed DCM, with a mean of 3 patient encounters per year. This compares to 2 encounters per year with patients with diagnosed DCM. The level of practitioner experience was positively correlated with the detection of undiagnosed DCM (P<.005). The influence of practitioner experience was corroborated by a subgroup analysis looking at the relationship between practitioner age on the detection of undiagnosed DCM. Osteopaths older than 54 years encountered an average of 4.2 cases per year, whereas those younger than 35 years detected 2.9 cases per year. Osteopaths in private clinics reported encounters with a greater mean number (4.4) of undiagnosed DCM cases per year than osteopaths in other clinic types (3.0).

**Conclusions:**

Osteopaths reported that they frequently consult people with DCM, including those suspected to have undiagnosed or presurgical DCM. Given this concentrated presentation of early DCM and a workforce professionally trained to examine musculoskeletal disease, osteopaths could have an important role in accelerating access to timely treatment. We included a decision support tool and specialist referral template as a tool to support onward care.

## Introduction

Degenerative cervical myelopathy (DCM) is an umbrella term for symptomatic spinal cord compression, secondary to a range of degenerative pathologies such as osteoarthritic or ligamentous changes of the cervical spine [1,2]. Symptoms are typically progressive and debilitating, typically resulting in permanent disability and poor quality of life [3,4].

DCM is the leading cause of spinal cord impairment worldwide [5]. Although the prevalence of surgically managed DCM is estimated to be 1.6 per 100,000 cases, this does not account for those managed nonoperatively or who do not obtain a diagnosis [6]. International guidelines emphasize that surgical decompression is the only evidence-based management for patients with moderate-to-severe or progressive DCM [7], and a more recent estimated based on imaging studies suggests that the overall prevalence could be 2.3% [8].

Although common, there is widespread underreporting of DCM [9]. Late diagnosis is typical, ultimately leading to increased morbidity, poorer quality of life [3,10,11], and an increase in the cost of care [9,12]. This problem is attributed in part to the heterogenous early features of the disease, including the loss of manual dexterity, neck pain [13,14], limb weakness, imbalance, gait impairment, and sensory disturbance [15]. This is compounded by a lack of awareness among many nonspecialist health care professionals [9,16].

Process mapping of the DCM diagnostic pathway in the United Kingdom has shown that specialist care is almost exclusively accessed via primary care, with patients visiting their general practitioner multiple times before referral to either musculoskeletal services or neurology [9,17-19]. Although primary care is therefore an attractive target for accelerating access to appropriate care, this is a challenging audience to reach. Primary care physicians remain under substantial pressure to remain up to date across the breadth of health care [20] and, on an individual basis, may see DCM infrequently. Although improving DCM education for all health care professionals is important [21], focusing efforts on concentrated triage points may be more efficient.

*Myelopathy.org*, an international DCM charity, is attempting to address this challenge, and through its community, has noted that many people with DCM [22] seek care from allied health care professionals, such as physiotherapists, chiropractors, and osteopaths. This is believed to occur both before and after diagnosis, although the frequency, nature, and outcomes of these interactions are poorly characterized. The objective of this work was therefore to investigate the nature and frequency of osteopathic care provided to individuals with DCM, to determine if osteopathy could serve as a focal point for improving timely access to appropriate treatment.

## Methods

### Survey Design

The DCM osteopathy survey was designed to capture current interaction between people with DCM and osteopaths in the United Kingdom and is reported following the Checklist for Reporting Results of Internet E-Surveys [23].

A cross-sectional observational study was conducted using a web-based survey targeted at practicing osteopaths.

Questions captured osteopath demographics including age, gender, ethnicity, the year of qualification, region of practice, type of practice, and Institute of Osteopathy (iO) membership status. The number of people with DCM encountered yearly by each osteopath was captured in the form of grouped data (0, 1-5, 6-10, 11-15, or 16+).

The questionnaire included questions to assess the frequency with which the following groups of patients with DCM are encountered by osteopaths (Multimedia Appendix 1):

- Diagnosed, without surgical intervention
- Diagnosed, following surgical intervention
- Undiagnosed

A case of undiagnosed DCM was determined based upon osteopath clinical assessment, including history and examination. Osteopaths determined a case of diagnosed DCM if either (1) the patient reported having received a clinical diagnosis of DCM, or (2) available written clinical records revealed a diagnosis of DCM.

### Ethical Considerations

According to the National Health Service (NHS) Health Research Authority, Research Ethics Committee approval was not required for this work.

The survey questions were approved by the iO for inclusion in their triennial practitioner census. The iO is the professional membership body for osteopaths in the United Kingdom, representing two-thirds of those practicing. Although their primary focus is the osteopath community, the iO also endeavors to engage with other health care professionals, agencies, and patient groups.

Participants completed the survey voluntarily and were informed before doing so that anonymized data would be shared with researchers at the University of Cambridge and the charity *Myelopathy.org* for the purposes of academic research. This acted as voluntary electronic consent, with the completion of the survey questions taken as agreement to participate.

Identifiable subject data were not required, and therefore, anonymized data were provided to members of our research team.

Entry into a prize draw to win a £100 (US $124.09) Amazon voucher was offered by the iO as an incentive to complete the survey.

### Development and Testing

The usability and technical functionality of the survey was piloted by iO before dissemination.

### Data Protection

No patient identifiable information was stored. The minimum amount of data was securely stored and accessed by the minimum number of researchers for the minimum amount of time required to complete the research.

### Participants

All participants were practicing osteopaths in the United Kingdom.

### Recruitment

A closed survey type was used, with the practitioner’s unique General Osteopathic Council registration number required to commence the survey. The iO census was advertised and distributed via the iO social media channels.

### Administration

The census was hosted on the iO website using Warp (Omnisis). As the iO is the primary professional body representing UK osteopaths, there was no inappropriate sample preselection. The survey was not administered via email. The completion of the survey was voluntary; however, an incentive of entry into a prize draw for a £100 (US $124.09) Amazon voucher was offered (Multimedia Appendix 2). Adaptive questioning was used to reduce complexity, and respondents were able to review their answers by using a “Back” button. Responses were collected from February 26, 2021, to May 1, 2021.

### Response Rates

In June 2021, there were 5410 osteopaths on the General Osteopathic Council register. As 571 participants completed the census, the minimum response rate was 10.6%.

Participants were required to enter their unique General Osteopathic Council registration number to commence the survey, preventing multiple entries from the same individual.

### Data Analysis

Survey data were extracted into an Excel spreadsheet (Microsoft). Analysis and data visualization were performed using R (version 4.0.5; R Foundation for Statistical Computing) and Rstudio (version 1.4.1106; Rstudio Team). Incomplete responses were excluded from the analysis.

When calculating the mean frequency of patient encounters, the group midpoint for 16+ encounters was taken to be 20.

## Results

### Summary

A total of 547 currently practicing osteopaths participated in the survey: 36.9% (n=202) were male and 63.1% (n=345) were female. This included osteopaths with all levels of experience: 206 (37.7%) qualified before 2000 and 341 (62.3%) after 2000, with 170 (31.1%) having been qualified since 2017. Similarly, there were responses from osteopaths of all age groups: <35 years (n=82, 15%), 35-44 years (n=126, 23%), 45-54 years (n=153, 28%), and >54 years (n=186, 34%). Although responses came from every region of the United Kingdom, 276 (50.5%) responses came from London and the Southeast, reflecting a high density of osteopathy clinics, compared to 281 (51.4%) in the rest of the United Kingdom. All practice types were represented, including sole practitioners (n=197, 36%), osteopathic private clinics (n=224, 41%), multidisciplinary private clinics (n=115, 21%), and NHS clinics (n=11, 2%). Current members of the iO accounted for 94% (n=514) of the responses. The average survey completion time of the 20-minute survey was 2 hours 28 minutes, suggesting completion over multiple sessions.

### DCM Survey Responses

At least 377 (68.9%) respondents reported an encounter with a patient with DCM each year. The modal number of patients with DCM encountered by an osteopath for the undiagnosed and diagnosed but not undergone surgical treatment groups was 1-5 per year. Approximately half of osteopaths (291/547, 53.2%) reported not encountering any patients with DCM who had undergone surgery, whereas approximately half (242/547, 44.2%) reported encountering 1-5 per year (Table 1).

Osteopaths most frequently encountered patients with undiagnosed DCM, with a mean of 3.0 patient encounters per year. This compares to 1.6 and 2.4 encounters per year with patients with DCM that had and had not undergone surgical intervention, respectively (Table 2).

**Table 1:** Summary table of responses to degenerative cervical myelopathy (DCM) questions. For a more detailed explanation of questions asked, see Multimedia Appendix 1 (N=547).

Number of patients encountered per year	Diagnosed DCM, but not undergone surgical treatment, n (%)	Diagnosed DCM who have undergone surgical treatment, n (%)	Undiagnosed DCM, n (%)
0	227 (41.5)	291 (53.1)	170 (31.1)
1-5	267 (48.8)	242 (44.2)	306 (55.9)
6-10	36 (6.6)	10 (1.8)	47 (8.6)
11-15	14 (2.6)	3 (0.5)	16 (2.9)
16+	3 (0.5)	1 (0.2)	8 (1.5)

**Table 2:** Mean number of encounters per year, showing that undiagnosed degenerative cervical myelopathy (DCM) is the most common group of patients with DCM to be encounters by osteopaths.

Type of patient encounter	Encounter per year, mean
Diagnosed DCM + no surgery	2.4
Diagnosed DCM + surgery	1.6
Undiagnosed DCM	3.0

### Practitioner Level of Experience

A subgroup analysis (Table 3) was conducted to assess the influence of practitioner experience on the detection of undiagnosed DCM. A positive correlation was suspected between an earlier year of qualification and the number of reported encounters with patients with undiagnosed DCM. The mean number of patient encounters was 3.6 for pre-2000 qualification, 2.7 for post-2000 qualification, and 1.9 for qualification since 2017.

A chi-square test of independence was conducted to investigate this association by comparing the observed frequencies of encounters in each category to the frequencies that would be expected under an assumption of independence. The results of the chi-square test indicated a statistically significant correlation (*P*<.005).

The influence of practitioner experience was corroborated by a subgroup analysis looking at the relationship between practitioner age on the detection of undiagnosed DCM (Table 4). Osteopaths older than 54 years encountered an average of 4.2 cases per year, whereas those younger than 35 years detected 2.9 cases per year.

**Table 3:** Number of encounters with patients with undiagnosed degenerative cervical myelopathy per year as a function of the year of qualification.

Number of encounters	Year qualified and responses				
	2017-2019 (n=70), n (%)	2018-2019 (n=57), n (%)	2019 (n=44), n (%)	Post-2000 (n=341), n (%)	Pre-2000 (n=206), n (%)
0	36 (51.4)	30 (52.6)	25 (56.8)	122 (35.8)	48 (23.3)
1-5	29 (41.4)	22 (38.6)	15 (34.1)	182 (53.4)	124 (60.2)
6-10	4 (5.7)	4 (7)	3 (6.8)	25 (7.3)	22 (10.7)
11-15	1 (1.4)	1 (1.8)	1 (2.3)	9 (2.6)	7 (3.4)
16+	0 (0)	0 (0)	0 (0)	3 (0.9)	5 (2.4)

**Table 4:** Number of encounters with patients with undiagnosed degenerative cervical myelopathy per year as a function of osteopath age.

Number of encounters	Osteopath age and responses			
	<35 years (n=82), n (%)	35-44 years (n=126), n (%)	45-54 years (n=151), n (%)	55+ years (n=188), n (%)
0	36 (43.9)	41 (32.5)	51 (33.8)	42 (22.3)
1-5	40 (48.8)	68 (54)	84 (55.6)	114 (60.6)
6-10	5 (6.1)	12 (9.5)	12 (7.9)	18 (9.6)
11-15	0 (0)	5 (4)	3 (2)	8 (4.3)
16+	1 (1.2)	0 (0)	1 (0.7)	6 (3.2)

### Practice Type

A further subgroup analysis (Table 5) was conducted to compare the detection of undiagnosed DCM in different practice types.

**Table 5:** Number of encounters with patients with undiagnosed degenerative cervical myelopathy per year as a function of type of practice.

Number of encounters	Practice type and responses			
	MD^a^ NHS^b^ service (n=14), n (%)	MD private clinic (n=271), n (%)	Osteopathic private clinic (n=138), n (%)	Sole practitioner (n=236), n (%)
0	2 (14.3)	90 (33.2)	38 (27.5)	78 (33.1)
1-5	8 (57.1)	148 (54.6)	81 (58.7)	130 (55.1)
6-10	3 (21.4)	21 (77.5)	14 (10.1)	17 (7.2)
11-15	1 (7.1)	9 (3.3)	4 (2.9)	5 (2.1)
16+	0 (0)	3 (1.1)	1 (0.7)	6 (2.5)

^a^MD: multidisciplinary.

^b^NHS: National Health Service.

There was a similar frequency of patient encounters in each practice type, with the exception of multidisciplinary NHS clinics. Osteopaths in these clinics reported a mean of 4.4 cases of undiagnosed DCM cases per year, compared to a mean of 3.0 cases per year in other clinic types.

A one-sample 2-tailed *t* test was conducted to compare the mean number of undiagnosed DCM cases detected by osteopaths in multidisciplinary NHS clinics (4.4 cases per year) to the mean in other clinic types (3.0 cases per year). The results revealed a significant difference between the 2 groups (*t*_2_=–16.028; *P*=.004), indicating that osteopaths in multidisciplinary NHS clinics reported a higher mean number of undiagnosed DCM cases per year.

## Discussion

### Principal Findings

Most osteopaths encounter people with DCM, especially those who have not yet received a formal diagnosis. The frequency of encounters with patients with undiagnosed DCM is the highest among the most experienced osteopaths and perhaps also for those that work in multidisciplinary NHS clinics. Osteopathy may therefore represent a professional body well positioned to support improved care in DCM.

One of the major challenges currently facing people with DCM is delayed diagnosis. For a progressive, largely irreversible condition with treatment available that can halt progression, early diagnosis is fundamentally important for good patient outcomes [24]. Unfortunately, there are currently significant diagnostic delays [1,9]. The current diagnostic pathway is poorly understood, and evidence of heterogeneity in referrals has demonstrated the lack of a unified pathway [17]. Addressing this was identified among the top 10 priorities for DCM by the AO Spine Research Objectives and Common Data Elements for DCM (RECODE-DCM) initiative, which was endorsed by the National Institute for Health Research (NIHR) James Lind Alliance [25].

The reported average of 3 consultations per year for undiagnosed DCM is noteworthy. Hospital Episode Statistics (NHS Digital) for England demonstrate that the NHS, over the last decade, has managed at most 5000 DCM cases per annum [26]. Although this figure represents secondary care and is thought to primarily reflect operative treatment, it is important to consider the potential implications of the reported average of 3 consultations per year for undiagnosed DCM among osteopaths. If this average were to be extrapolated cautiously across all 5410 registered osteopaths, it would suggest a considerably larger number of DCM cases encountered in the primary care setting.

The origins of osteopathy lie in the manipulation of bodily tissues, such as bone and muscle, to treat a range of different symptoms [27]. The founding principal was that these manipulations could improve blood flow and, therefore, promote healing [28]. This approach is distinct to that of chiropractors, which a separate profession, who aim to use manipulation to alleviate neural compression. Osteopathy today is a regulated medical profession, subject to professional qualifications [29]. In fact, within the United Kingdom, osteopaths train for a minimum of 4 years to reach degree level. Osteopaths are primarily therefore musculoskeletal practitioners, offering a multimodal package of care (eg, manual therapy, psychological support, and exercise) to provide patient-centered management of pain and enable self-management. Some osteopathy services are currently commissioned by the NHS in the United Kingdom. Although the mechanistic basis of osteopathy may remain uncertain, there are high-quality studies demonstrating noninferiority to standard care [30], and for musculoskeletal symptoms such as pain, where medical approaches are moving away from the limitations of a Cartesian model, these therapies may align with more contemporary and holistic approaches to symptom management [31-33].

Consequently, as osteopaths are specifically trained to assess and manage musculoskeletal conditions and people with undiagnosed DCM may be likely to consult them, the osteopath community may be well suited to supporting earlier diagnosis and improving access to timely treatment. Within the UK health system, as most osteopaths operate in the private sector, this would require a means of integrating with NHS pathways. To this end, a template referral letter has been co-designed by *Myelopathy.org* to help osteopaths prompt general practitioners to consider this diagnosis and initiate appropriate investigation (Multimedia Appendix 3).

Although spinal cord compression in DCM has traditionally been conceptualized as a static spinal cord injury, recent thinking suggests an additional component of dynamic injury [34]. Certain manipulations of the spine may therefore risk further injury to the spinal cord in some patients [35]. Nonoperative management of DCM also remains as a significant knowledge gap for further research [7]. Although broad efforts are being undertaken to raise awareness of DCM among clinicians and patients [10,22,36], targeted education and training among specific groups, who may use such techniques, could have benefits beyond accelerating diagnosis.

### Limitations and Future Directions

This survey was designed to capture the professional perspective from osteopaths, and the patient perspective is also needed. The survey results may be subject to recall bias, and further questions were not included to ascertain how specifically the DCM diagnostic status was confirmed.

In the subgroup analysis, there is an underlying assumption that the clinical suspicion of senior osteopaths is more accurate than that of their junior colleagues. It is also assumed that they are consulting comparable patient populations. These assumptions may be unfounded.

In addition, the findings of the subgroup analysis of practice type are likely to be insignificant due to a very small sample of NHS osteopaths.

The theoretical minimum response rate of 10.6% is highly likely to be a significant underestimate given that not all osteopaths would be expected to engage with the iO website and not all those who do would necessarily be aware of the census.

Nonetheless, our survey captures data from a large number of osteopaths from a wide range of professional backgrounds and represents the first published description of this interaction from the United Kingdom.

### Conclusions

Osteopaths reported that they frequently consult people with DCM, including those suspected to have undiagnosed or presurgical DCM. Given this concentrated presentation of early DCM and a workforce professionally trained to examine musculoskeletal disease, osteopaths could have an important role in accelerating access to timely treatment.

## Appendix

Multimedia Appendix 1 DCM osteopathy survey. iO: Institute of Osteopathy; DCM: degenerative cervical myelopathy.

Multimedia Appendix 2 Osteopathy census.

Multimedia Appendix 3 Template referral letter.

## Abbreviations

DCM: degenerative cervical myelopathy
iO: Institute of Osteopathy
NHS: National Health Service
NIHR: National Institute for Health Research
RECODE-DCM: Research Objectives and Common Data Elements for Degenerative Cervical Myelopathy

## Data Availability

The data sets generated during and/or analyzed during the current study are available from the corresponding author on reasonable request.
