# Supplementary figures and images for: Osteopathy in the Early Diagnosis and Management of Degenerative Cervical Myelopathy: National Survey
Source: JMIR Form Res. 2023 May 9;7:e45248. doi: 10.2196/45248 (PMC10206621; doi:10.2196/45248)

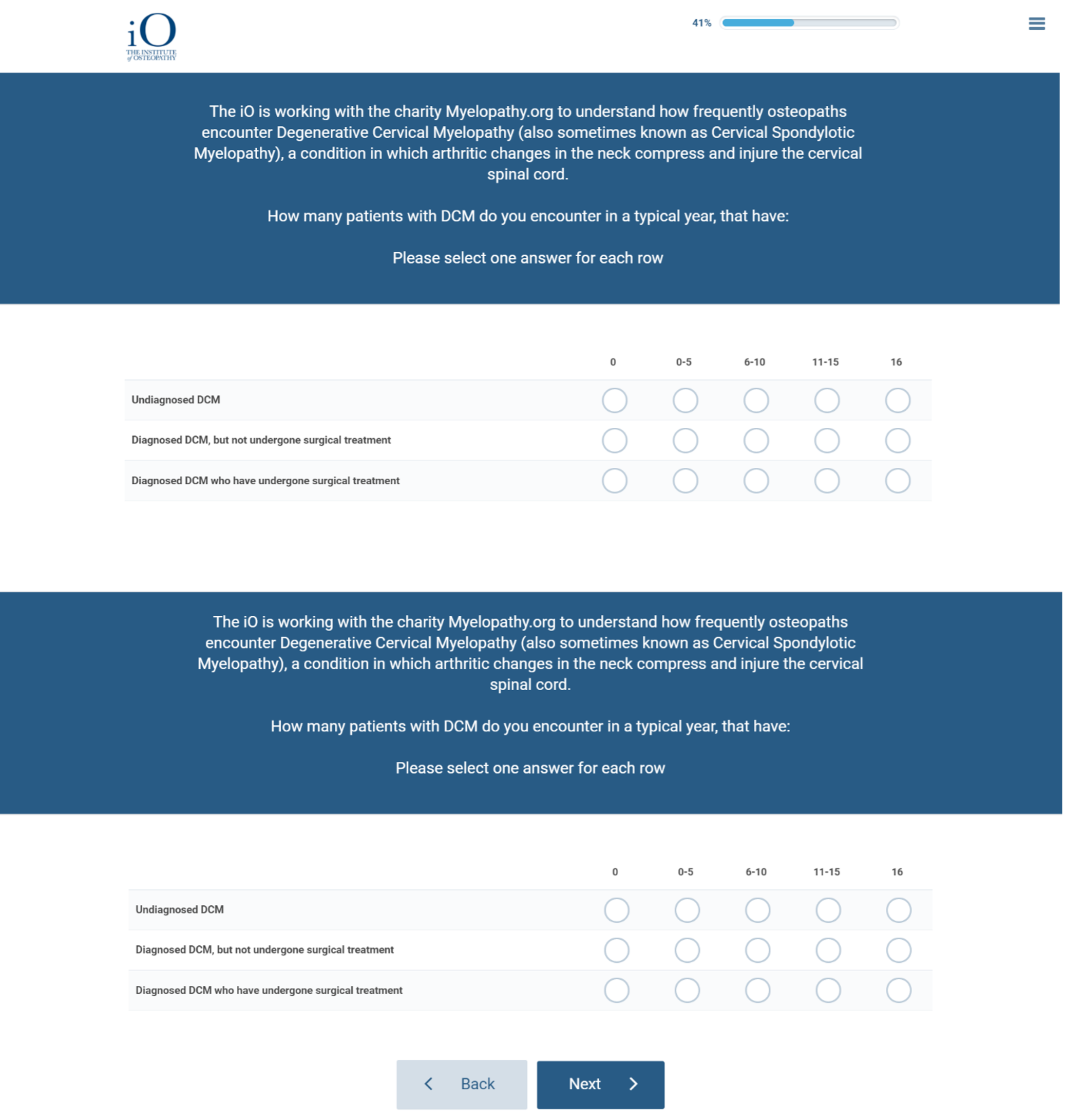

Supplement: Multimedia Appendix 1 [file formative_v7i1e45248_app1.png]

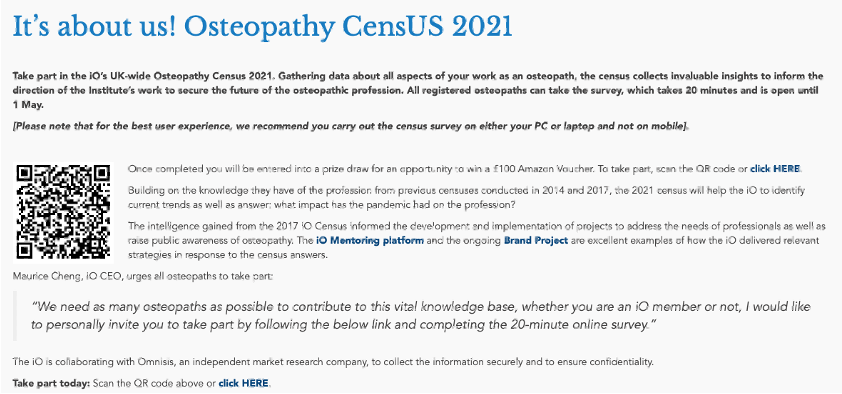

Supplement: Multimedia Appendix 2 [file formative_v7i1e45248_app2.png]

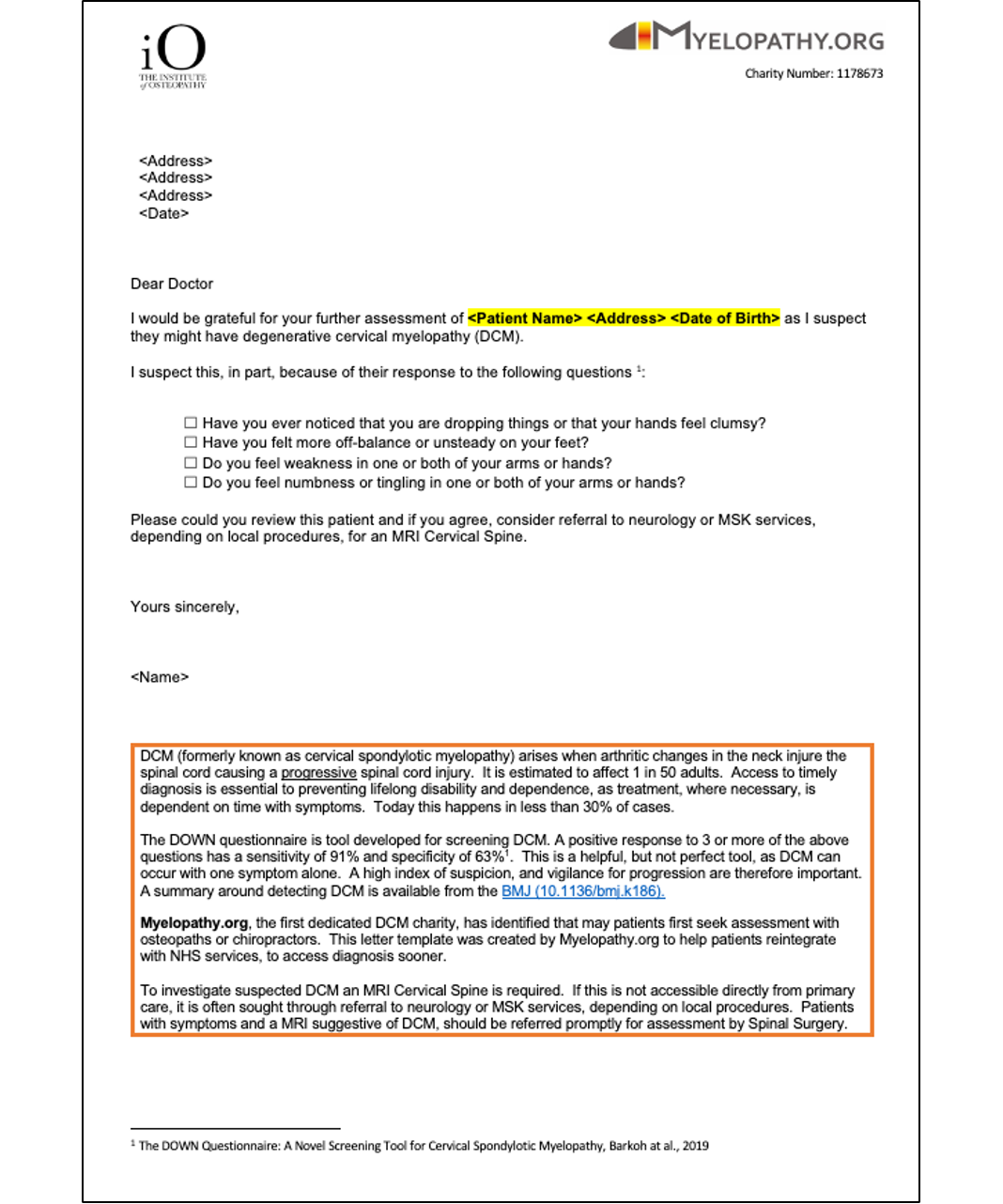

Supplement: Multimedia Appendix 3 [file formative_v7i1e45248_app3.png]
